# Randomized trial of nicotine replacement therapy (NRT), bupropion and NRT plus bupropion for smoking cessation: effectiveness in clinical practice

**DOI:** 10.1111/add.12304

**Published:** 2013-08-28

**Authors:** John Stapleton, Robert West, Peter Hajek, Jenny Wheeler, Eleni Vangeli, Zeinab Abdi, Colin O’Gara, Hayden McRobbie, Kirsty Humphrey, Rachel Ali, John Strang, Gay Sutherland

**Affiliations:** 1 Addictions Department, Institute of Psychiatry, Kings College LondonLondon, UK; 2 Cancer Department of Epidemiology and Public Health, Research UK Health Behaviour Research Centre, University College LondonUK; 3 Wolfson Institute of Preventive Medicine, Barts and The London School of Medicine and Dentistry, Queen Mary Univesity of LondonLondon, UK; 4 Mid-Essex PCTEssex, UK; 5 St. John of God HospitalStillorgan, Dublin, Ireland; 6 Central and North West London NHS TrustLondon, UK; 7 Addictions Directorate, South London and Maudsley Hospital Foundation TrustLondon, UK

**Keywords:** Bupropion, combination treatment, depression, NHS, NRT, randomized trial, smoking cessation, varenicline

## Abstract

**Background and aims:**

Bupropion was introduced for smoking cessation following a pivotal trial showing that it gave improved efficacy over the nicotine patch and also suggesting combination treatment was beneficial. We tested in clinical practice for an effectiveness difference between bupropion and nicotine replacement therapy (NRT), whether the combination improves effectiveness and whether either treatment might be more beneficial for certain subgroups of smokers.

**Design:**

Open-label randomized controlled trial with 6-month follow-up.

**Setting:**

Four UK National Health Service (NHS) smoking cessation clinics.

**Participants:**

Smokers (*n* = 1071) received seven weekly behavioural support sessions and were randomized to an NRT product of their choice (*n* = 418), bupropion (*n* = 409) or NRT plus bupropion (*n* = 244).

**Measures:**

The primary outcome was self-reported cessation over 6 months, with biochemical verification at 1 and 6 months. Also measured were baseline demographics, health history, smoking characteristics and unwanted events during treatment.

**Findings:**

Abstinence rates for bupropion (27.9%) and NRT (24.2%) were not significantly different (odds ratio = 1.21, 95% confidence interval = 0.883–1.67), and the combination rate (24.2%) was similar to that for either treatment alone. There was some evidence that the relative effectiveness of bupropion and NRT differed according to depression (χ^2^ = 2.86, *P* = 0.091), with bupropion appearing more beneficial than NRT in those with a history of depression (29.8 versus 18.5%). Several unwanted symptoms were more common with bupropion.

**Conclusion:**

There is no difference in smoking cessation effectiveness among bupropion, nicotine replacement therapy and their combination when used with behavioural support in clinical practice. There is some evidence that bupropion is more beneficial than nicotine replacement therapy for smokers with a history of depression.

## Introduction

The antidepressant bupropion hydrochloride SR (Zyban; GlaxoSmithKline, London, UK) was introduced as the first non-nicotine medicine for smoking cessation following a manufacturer-sponsored Phase III trial 1. The main result showed bupropion to be substantially more beneficial than a nicotine patch, the most widely used type of nicotine replacement therapy (NRT) [odds ratio (OR) = 2.07, 95% confidence interval (CI) = 1.22–3.53]. In combination, bupropion and the patch were superior to the patch alone, although not compared with bupropion alone.

Despite the importance of this primary result, only two small trials have since compared bupropion with NRT, both failing to show a difference 2,3. When data from the three trials are summarized, the result is equivocal because of a suggestion of heterogeneity and uncertainty about the most appropriate pooling model 4. Bupropion is more effective using fixed-effect modelling (OR = 1.54, 95% CI = 1.01–2.37), but not with a random-effect model (OR = 1.34, 95% CI = 0.71–2.56). There has been more interest in combination treatment, with five subsequent small trials all failing to find a benefit for bupropion plus NRT compared with NRT alone, albeit with widely different study populations and smoking phenotypes 5–9. In all six trials there is no evidence of benefit (OR = 1.29, 95% CI = 0.63–2.62—author random-effects meta-analysis). No trials have since tested the combination against bupropion alone, arguably a more relevant comparison given that the evidence points towards bupropion being more effective than NRT.

With much uncertainty remaining, we conducted an effectiveness trial of these therapies in a clinical practice setting. Three main questions were addressed: (i) is there a difference in effectiveness between NRT and bupropion; (ii) is the combination superior to either treatment alone; and (iii) can subgroups be identified who would benefit more from one or other treatment?

## Methods

### Study overview

This was an investigator-led trial conducted in four National Health Service (NHS) smoking cessation services, sited at the Maudsley Hospital, London (*n* = 216), the Royal London Hospital (*n* = 276), the South-East Essex Stop Smoking Service (*n* = 321) and the Haringey and Enfield Stop Smoking Service, London (*n* = 258). Local research ethics approval was received for each site. The UK Medical Research Council oversaw management, with funding provided by the Department of Health for England. The trial was sponsored by Kings College London and authorized by the Medicines and Healthcare products Regulatory Agency (MHRA). All participants gave informed consent. The first participant enrolled on 15 June 2004 and the database was completed on 30 September 2007.

### Study design

This was an open-label parallel-group trial with participants randomized to NRT or bupropion or NRT plus bupropion. Given the established efficacy of NRT and bupropion as single therapies, a group receiving no treatment or a placebo was considered unethical and impracticable. Because all medicines had been used as equal first-line treatments for several years in the NHS, an open-label design was not considered likely to induce a bias in favour of a particular treatment.

Randomization was in the ratio 7 : 7 : 4 for NRT, bupropion and the combination, respectively, to reflect the importance of the main comparison between NRT and bupropion alone. Behavioural support was provided in the usual style at each clinic, involving seven weekly, mainly group support, sessions (each 60–90 min) over 6 weeks: (i) assessment/screening, (ii) preparation, (iii) quit-day, (iv) week 1 post-quit, (v) week 2 post-quit, (vi) week 3 post-quit and (vii) week 4 post-quit 10,11. Trial participants followed the same procedures and treatment protocol as usual-care smokers, the only additions being random assignment to treatments and follow-up at 6 months.

Following usual clinical practice, smokers contacting each clinic for help were booked to attend the next treatment course at their convenience. An information pack was sent out before the first session. This included a self-completion questionnaire to aid assessment and a letter explaining the trial and inviting them to participate. Between 10 and 30 smokers attended each course, depending on demand at the time. At the first session individual assessment preceded information in a group on the treatment options offered. Those interested in trial participation were screened individually for eligibility and, depending on the preference of each clinic, consent and randomization took place either at this time or at the next (pre-quit) session, 1 week later.

Participants received cessation support in groups that included smokers who had not enrolled in the trial. Session 2 (preparation) consisted of therapist guidance and group discussion in preparation for quit day. Those taking bupropion started their medication after this session. Those using NRT started treatment from session 3 (quit day) and all participants were encouraged to stop smoking completely from that day onwards. Weekly support was given at sessions 4–7 (1–4 weeks after quit day). Those failing to attend the final session were contacted and encouraged to attend at a later time. Three attempts were made to contact all participants 26 weeks later, and those reporting not having smoked during the previous 7 days were invited to attend for a brief follow-up assessment for which travelling expenses were reimbursed. Blind follow-ups were not considered feasible, due to patients usually discussing their treatment when contacted.

### Study sample

Participants were daily smokers who did not express a preference for a particular treatment, were able to understand the trial procedures, were willing to participate, and for whom neither NRT nor bupropion were contraindicated according to the summary of product characteristics (SpC).

### Study medication

Study medication was provided free of charge to the trial in standard packaging by all the manufacturers (Pfizer UK, GSK UK, Novartis UK). NRT was dispensed by trial clinicians/pharmacists under whichever scheme operated locally. At two sites with prescribing doctors, bupropion was dispensed under arrangement with the local pharmacy. At the two sites without doctors, bupropion was prescribed in the usual way by the participant's primary care doctor [general practitioner (GP)], following screening at the clinic. GPs were asked to adhere to the randomized treatment assignment.

Following usual practice in NHS clinics, participants assigned to NRT could choose a single product, after consultation with clinic staff regarding such considerations as previous experience with products and appropriate dosage. Dosage over the 12-week course could be adjusted or the product type changed as considered necessary. Participants assigned to bupropion took 150 mg bupropion for the first 6 days and 300 mg for the remainder of the 8-week course, in line with the SpC. The dose could be reduced if considered necessary and those experiencing unacceptable symptoms could stop taking bupropion and switch to NRT. Following NHS practice, medication was dispensed to participants in batches over time 12. It was usually given every 2 weeks throughout the support phase until the final session (session 7) when the remainder of the course was dispensed to those continuing.

### Study procedures and measures

To allow participants to be recruited at satellite centres without computer links, randomization was by sealed envelope. Numbered envelopes containing a random treatment allocation were provided to each site. On enrolment participants selected their envelope from a large batch and signed it before breaking the seal to reveal their allocation. Randomization and packaging was organized by an independent statistician at the host site, using the SPSS software *Rand* function. The sites received batches of 90 envelopes as required throughout recruitment, each consisting of five blocks of 18 random assignments (seven NRT, seven bupropion, four NRT + bupropion).

Participants completed the usual clinic materials. The pre-treatment questionnaire included items relating to demographics, smoking history, tobacco dependence, previous attempts to stop and medical history 13. It was self-completed and responses were reviewed with a clinician at the first session. At the start of sessions 2–7, participants completed a form detailing smoking behaviour and use of medications throughout the previous week. At session 2 this included ratings of mood effects and from session 3 onwards a section where up to three unwanted symptoms could be reported. When reported, the intensity of the symptom was self-rated: 1 = mild, 2 = moderate, 3 = severe. Before each support session a therapist reviewed the responses and recorded an expired-air carbon monoxide measure (ECO). At 6-month follow-up participants completed a questionnaire detailing their smoking behaviour since the last support session and an ECO was taken.

### Outcome measures and data analysis

The primary outcome measure followed the Russell Standard criterion for 6 months cessation (RS6) 14, defined as a self-report not having smoked more than five cigarettes between the end of the second week post-quit (session 5) and the 6-month follow-up (including complete abstinence during weeks 3 and 4 post-quit and during the final week of follow-up), and recording an ECO level of <10 parts per million (p.p.m.) at session 7 and at 6 months. Hence, patients needed to self-report 2 weeks of abstinence and be validated at session 7 (end of treatment) and to report 6 months of abstinence and be validated at the 6-month follow-up, with the allowance of only five cigarettes having been smoked over the follow-up period. Secondary measures were point-prevalence at 6 months (PP6), defined as a self-report of completed abstinence during the final week of follow-up and an ECO level <10 p.p.m., and the Department of Health short-term monitoring measure (DH4), defined as a self-report of complete abstinence during weeks 3 and 4 post-quit and an ECO level <10 p.p.m. at session 7. By definition, all those classified as RS6 abstinent were also DH4 and PP6 abstinent. Those not attending session 7 or at 6 months were considered as continuing smokers, or to have relapsed 14,15.

The study was designed to detect as significant an odds ratio of 1.5 for bupropion versus NRT which, based on an anticipated 25% 6-month cessation rate with NRT (34% bupropion) and with alpha and beta (1-power) set at 0.05, would require sample sizes of 700 (NRT) and 700 (bupropion). Given the weaker prior evidence for the combination compared with bupropion, 0.90 power was considered appropriate for this comparison (i.e. 44 versus 34%), requiring an additional 400 participants in a combination group (1800 participants in total). In the event, recruitment was slower than anticipated over the time clinics allocated and the trial closed with 1071 participants (418 NRT, 409 bupropion, 244 combination). It remained considerably larger than the manufacturer trial 1, and had 0.90 power to detect an odds ratio of 1.6 for bupropion versus NRT (i.e. 35% versus 25%).

Data were entered initially into local databases before transfer to a database at the lead site. Smoking cessation rates were compared using the OR with 95% CI, and logistic regression models were used to adjust for potentially confounding antecedent characteristics. We examined whether there were subgroups who benefited particularly from either NRT or bupropion by fitting logistic regression models with interaction terms for treatment by each of the characteristics of interest. The trial protocol specified that if there was no difference between single treatments, pooled data would be used for comparison with combination treatment. Unwanted symptom incidence and intensity were compared using χ^2^ tests. Data were processed and examined only after all follow-ups had been completed and the data file closed.

## Results

### Characteristics of the participants

Table 1 shows the participant characteristics by treatment arm. Overall, 47% were male and the mean age was 41 years. They smoked an average 20 cigarettes per day (range three to 60). About 40% reported a history of a major illness associated with smoking and more than a quarter reported a history of depression. Approximately 60 and 6%, respectively, had used NRT and bupropion previously, and 38% had succeeded in stopping smoking for as long as 1 month in the previous 5 years. In those assigned to NRT, 48% (201 of 418) chose to use the patch, with approximately equal numbers using the other five product types (gums, lozenges, inhalator, nasal spray and microtab).

**Table 1:** Characteristics of participants on entry to the trial

	NRT (n *=* 418)	Bupropion (n *=* 409)	NRT and bupropion (n *=* 244)
Demographics			
% Male (*n*)	47.8 (200)	45.5 (186)	47.0 (115)
Age, mean (SD)	40.8 (11.9)	40.7 (11.7)	41.2 (12.1)
% White European origin (*n*)	78.2 (327)	78.5 (321)	79.5 (194)
% No school or college qualifications (*n*)	23.2 (97)	26.4 (108)	20.1 (49)
% Receiving state benefits (*n*)	33.3 (139)	37.2 (152)	38.1 (93)
Health			
% Life-time history of major illness related to smoking (*n*)	37.1 (155)	40.1 (164)	42.6 (104)
% Life-time history of depression (*n*)	25.8 (108)	25.7 (105)	27.0 (66)
% Life-time history of drug or alcohol problems (*n*)	4.55 (19)	3.91 (16)	4.51 (11)
Tobacco smoking			
Usual daily cigarettes smoked, mean (SD)	20.7 (8.7)	19.8 (8.1)	20.3 (9.7)
HSI score, mean (SD)^a^	3.26 (1.41)	3.13 (1.48)	3.28 (1.47)
% ‘Very’ or ‘totally’ determined to stop at this attempt (*n*)	77.3 (323)	77.8 (318)	71.7 (175)
Confidence in stopping at this attempt (1–10), mean (SD)^b^	7.49 (2.02)	7.59 (1.93)	7.29 (1.97)
% Stopped for more than 1 month in last 5 years (*n*)	37.1 (155)	38.1 (156)	38.5 (94)
% Previously failed with NRT (*n*)	59.1 (247)	61.1 (250)	61.5 (150)
% Previously failed with bupropion (*n*)	7.42 (31)	4.16 (17)	8.20 (20)

a Heaviness of Smoking Index (HIS) scored as 0 (light)—6 (heavy), four subjects not recorded

b 69 participants not recorded. There was no detectable evidence of chance imbalances in the distributions of any of the characteristics shown when these were compared between the three cohorts (all *P* > 0.05). NRT = nicotine replacement therapy; SD = standard deviation.

**Figure 1:**
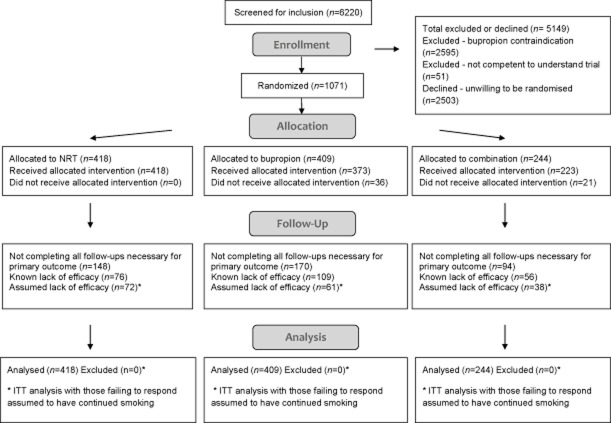
Flow of participants through the trial

Figure 1 shows the flow of participants through the trial 16. Those not enrolled were divided approximately equally between those having a treatment preference and therefore being unwilling to be randomized (*n* = 2503) and those for whom bupropion was contraindicated (*n* = 2595). In the two sites where local GPs were required to prescribe bupropion, 57 participants were refused prescriptions. The reported reasons appeared to be mainly unrelated to contraindications or cautions and were due either to the GP misunderstanding these or possibly having an individual prejudice against bupropion. In 27 of these cases the participant either switched to NRT (21 of 36) or continued NRT alone (six of 21). The remaining 20 participants preferred to continue without medication. The two follow-ups for the primary outcome (session 7, 26 weeks) were completed by 659 (61.5%) participants and this rate did not differ significantly between treatment groups (χ^2^_(2)_ = 3.33, *P* = 0.19). Of the 412 participants not completing both follow-ups, 241 (58.5%) reported smoking at one follow-up which they did complete, meaning that they could not be classified as RS6 abstinent. Hence, only for the remaining 171 (16.0% of all participants) who either did not respond to either follow-up or who were abstinent at one follow-up, but did not respond to the other, was it was necessary to assume continued smoking. This gave a primary outcome completion rate of 84%. Again, this rate did not differ between treatment groups.

### Outcomes

Abstinence rates were compared in the full sample as randomized (intention-to-treat—ITT sample) and in the sample excluding the 57 participants known not to have received their assigned treatment (treated sample). There was no evidence of an effectiveness difference between bupropion and NRT on any primary or secondary outcome measure in either the ITT or treated samples (Table 2). Although the OR appeared to increase in favour of bupropion between the short-term (DH4) and RS6 outcomes, relapse rates between these times were not significantly different (58.9% NRT, 50.5% bupropion; difference = 8.5%, 95% CI = −0.5 to 17.9%). There was also no evidence that compared with either single treatment, or the two single-treatment groups pooled together, that combination treatment was more effective.

**Table 2:** Smoking cessation outcome

Outcome measure	NRT	Bupropion	Combination	Odds ratio bupropion versus NRT (95% CI)	Odds ratio combination versus pooled NRT and bupropion (95% CI)
ITT sample^a^	(*n* = 418)	(*n* = 409)	(*n* = 244)		
Primary outcome (RS6) % (*n*)	24.2 (101)	26.7 (109)	23.4 (57)	1.14 (0.834–1.56)	0.896 (0.640–1.25)
Secondary outcome (DH4) % (*n*)	58.9 (246)	53.8 (220)	54.5 (133)	0.814 (0.618–1.07)	0.928 (0.697–1.24)
Secondary outcome (PP6) % (*n*)	28.2 (118)	32.3 (132)	27.5 (67)	1.21 (0.900–1.63)	0.874 (0.636–1.20)
Treated sample^b^	(*n* = 418)	(*n* = 373)	(*n* = 223)		
Primary outcome (RS6) % (*n*)	24.2 (101)	27.9 (104)	24.2 (54)	1.21 (0.883–1.67)	0.913 (0.647–1.29)
Secondary outcome (DH4) % (*n*)	58.9 (246)	54.4 (203)	55.6 (124)	0.835 (0.63–1.11)	0.954 (0.707–1.29)
Secondary outcome (PP6) % (*n*)	28.2 (118)	33.8 (126)	28.7 (64)	1.30 (0.959–1.76)	0.902 (0.651–1.25)

RS6 = Russell Standard sustained 6-month carbon monoxide (CO)-verified cessation; DH4 = Department of Health weeks 3 and 4 post-quit day CO-verified cessation; PP6 = 7-day CO-verified point prevalence at 6 months.

a Intention-to-treat (ITT)—sample as randomized.

b Treated sample—excluding 57 participants known not to have received bupropion. CI = confidence interval; NRT = nicotine replacement therapy.

Although there was evidence that RS6 abstinence rates differed between the four study sites (χ^2^_(3)_ = 13.7, *P* = 0.003), there was no evidence of a differential treatment effect (heterogeneity) across sites (χ^2^_(3)_ = 1.24, *P* = 0.745), or between the two sites who dispensed bupropion directly and the two sites which relied upon local GPs for bupropion prescriptions (χ^2^_(1)_ = 0.372, *P* = 0.542). Effect sizes were only marginally altered in logistic regression models that adjusted for all the baseline characteristics shown in Table 1. Among those assigned to NRT, RS6 success rates were similar between the six product types (χ^2^_(5)_ = 6.6, *P* = 0.247), with 26.9% of patch users (versus 26.7% bupropion) being successful.

### Subgroup analyses

**Figure 2:**
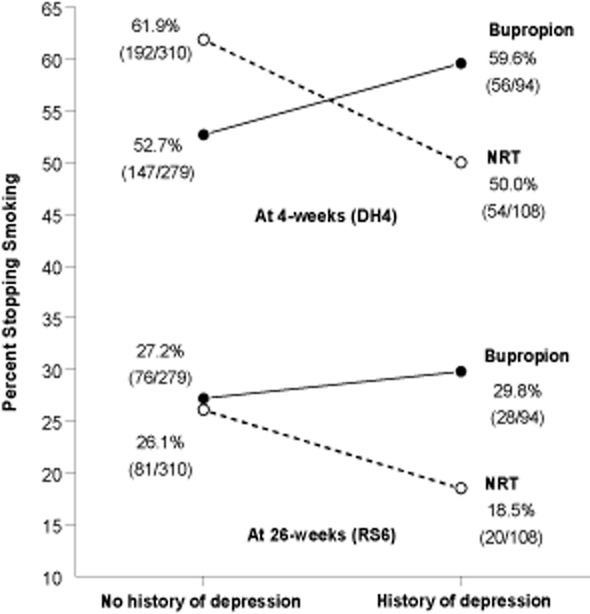
Relation between treatment, history of depression and smoking cessation at 4 and 26 weeks after quit day. NRT = nicotine replacement therapy

To examine if the effect of treatment was moderated for subgroups we fitted logistic regression models with interaction terms for treatment by each of the characteristics shown in Table 1. For this analysis we included only the 1014 participants known to have received their assigned treatment. Among these characteristics, only for life-time history of depression was there some evidence of a differential treatment effect (χ^2^ = 6.5, *P* = 0.011 and χ^2^ = 2.86, *P* = 0.091 for DH4 and RS6, respectively). This can be interpreted as there being evidence that bupropion was more effective than NRT for those with a history of depression (RS6: 29.8 versus 18.5%, χ^2^ = 3.523, *P* = 0.061) and there being no difference between treatments for those without a history of depression (Fig. 2). Alternatively, for those using NRT, a depression history was detrimental to success (DH4: *P* = 0.03; RS6: *P* = 0.075), whereas for those taking bupropion this effect was ameliorated (DH4: *P* = 0.246; RS6: *P* = 0.630). In all subjects, if treatment and interaction parameters are ignored, a history of depression did not affect the likelihood of success (DH4: *P* = 0.443; RS6: *P* = 0.418).

### Unwanted symptoms and use of medication

There were three cases of allergic reaction to bupropion, two of which resulted in anaphylaxis and required hospital admission. A fourth participant reported tearfulness and transient suicidal thoughts. These cases, plus one case of severe chest pain, were withdrawn from bupropion and offered NRT to continue their quit attempt. There were no such serious adverse events in the NRT group. Table 3 shows the incidence of non-serious unwanted symptoms as described by participants on the weekly forms after the commencement of treatment at sessions 3–7. Disturbed sleep was the most common symptom and experienced by more than 30% of those taking bupropion. Incidences of dry mouth, headache, nausea, dizziness, low mood/depression, anxiety/panic, chest pain, disorientation and loss of appetite were also higher among those taking bupropion. Nasal irritation (nicotine nasal spray) and skin irritation (nicotine patch) were more common among those using NRT. Among those experiencing symptoms, ratings of intensity were not significantly different between treatments. At session 2, before medication was started, there were no differences (all *P* > 0.05, data not shown) between the treatment conditions in the incidence of any of the mood effects similar to those reported later as unwanted symptoms (depression, irritability, restlessness, disturbed sleep, appetite).

**Table 3:** Unwanted symptoms after the commencement of treatment.^a^

Symptom	NRT (n *=* 418)	Bupropion (n *=* 409)	NRT plus bupropion (n *=* 244)
	Incidence n (%)^b^	Incidence n (%)^b^	Incidence n (%)^b^
Disturbed sleep	53 (12.7)	125 (30.6)^c^	76 (31.1)^d^
Vivid dreams	30 (7.2)	22 (5.4)	12 (4.9)
Skin irritation	30 (7.2)^c^,^d^	11 (2.7)	8 (3.3)
Sore throat/mouth	29 (6.9)	31 (7.6)	22 (9.0)
Headache	18 (4.3)	44 (10.8)^c^	21 (8.6)^d^
Bad taste	16 (3.8)	26 (6.4)	13 (5.3)
Irritation/agitation	16 (3.8)	17 (4.2)	18 (7.4)
Constipation	15 (3.6)	24 (5.9)	14 (5.7)
Nasal irritation	13 (3.1)^c^	3 (0.7)	3 (1.2)
Dry mouth	10 (2.4)	52 (12.7)^c^	30 (12.3)^d^
Nausea	9 (2.2)	24 (5.9)^c^	21 (8.6)^d^
Dizziness/lightheaded	5 (1.2)	22 (5.4)^c^	14 (5.7)^d^
Low mood/depression	2 (0.5)	17 (4.2)^c^	8 (3.3)
Chest pain	2 (0.5)	9 (2.2)^c^	5 (2.0)
Disorientated/confusion	2 (0.5)	7 (1.7)	7 (2.9)^d^
Loss of appetite	1 (0.2)	7 (1.7)^c^	6 (2.5)^d^
Anxiety/panic	1 (0.2)	12 (2.9)^c^	5 (2.0)

a Symptoms reported significantly more in comparisons or by at least 5% of participants in any treatment group, ordered by incidence in nicotine replacement therapy (NRT) cohort.

b Self-rated as moderate or severe in intensity at any time throughout treatment.

c Treatment with higher incidence, bupropion versus NRT (χ^2^, *P* < 0.05).

d Treatment with higher incidence, combination versus NRT (χ^2^, *P* < 0.05).

Use of medication was assessed among those abstinent (DH4) at 4 weeks (session 7). Seventy-six per cent (187 of 246) of those assigned NRT, compared with 63.1% (128 of 203) of those assigned bupropion, were continuing to use their medication on 5 or more days per week (χ^2^ = 8.9, *P* = 0.003). Of those assigned bupropion, 20.2% (41 of 203) had disliked the symptoms they attributed to it sufficiently to switch to NRT. Similarly, among those assigned combination treatment, 71 and 61% were using NRT and bupropion, respectively, on 5 or more days per week at this time.

## Discussion

We did not find evidence of an effectiveness difference between NRT, bupropion and their combination. With particular reference to the original manufacturer trial 1, we found no evidence of a difference between the nicotine patch and bupropion, although this comparison was not planned and involved self-selection of the nicotine patch in preference to other NRT products. Consequently, our results did not generalize to clinical practice the original efficacy findings of that trial with regard to the main comparison between bupropion and NRT, and were instead consistent with two other smaller efficacy trials 2,3. The only clear similarity in findings between ours and the original trial was that both reported a higher incidence of unwanted symptoms among those taking bupropion.

Given the divergence of findings comparing NRT and bupropion, evidence from the indirect comparison of numerous trials separately assessing NRT and bupropion should be considered. From 132 trials comparing any type of NRT with placebo or no treatment, the relative rate is 1.58 (95% CI = 1.50–1.66) 17. From 36 bupropion trials the equivalent rate is 1.69 (95% CI = 1.53–1.85) 4. The ratio of these two rates (1.69/1.58 = 1.07) is extremely close to that observed in the current trial (i.e. 26.7/24.2% = 1.10) and not distinguishable from 1. Therefore, the indirect comparison supports our finding rather than those of the original trial and its higher rate of 2.07 (1.2–3.5).

The reason why the manufacturer trial gave results at odds with subsequent findings cannot be discerned clearly. Regarding the comparison with our trial, an important design difference was our allowance for participants to select between all NRT products, rather than using only the nicotine patch. Although this feature models clinical practice more closely and the need for clinics to encourage smokers into treatment by being responsive to their preferences, it is unlikely to have boosted the NRT success rate sufficiently to have accounted for the difference. To date, there is no clear evidence of a difference in effectiveness between different NRT products or that individuals can select optimal products 17. It is possible that the strict inclusion/exclusion criteria in the manufacturer trial played a part, and that the far less restrictive criteria currently recommended for clinical practice allowed too many to use bupropion for whom it was unsuitable. This is possibly supported by our significantly poorer level of compliance with bupropion than NRT among those abstinent at 4 weeks. Although no specific data on compliance were given in Jorenby *et al*., the dropout rates published probably suggest similar levels of compliance in that trial.

Our trial had many of the limitations inherent in effectiveness trials conducted in routine clinical practice. Principally, we could not include placebos to allow a no-treatment condition or to blind treatment allocation and, although we waited until all treatments had long become routine before conducting the trial, preference bias remains a possibility. We were also unable to assess medication compliance comprehensively due to patients failing to attend or respond to enquiries. Additionally, we were unable to achieve the designed sample size, although lack of statistical power is unlikely to have been a serious limitation. If our estimated bupropion versus NRT difference approximates the true difference, as supported by an indirect comparison, a sample size of approximately 10 000 would be required to detect this as statistically significant. More importantly, it would probably be of negligible clinical significance.

With these findings in mind, it is worth considering the position of bupropion for smoking cessation. Bupropion efficacy appears to be lower than for varenicline 18, and while apparently similarly as effective as NRT, it causes a higher incidence of some unwanted effects (nine effects noted in this study as against two effects with higher NRT incidence). Might unwanted events be reduced without loss of efficacy with 150 rather than 300 mg bupropion? The original dose-ranging trial would suggest so 19. Longer-term cessation rates for 150- and 300-mg doses were indistinguishable, while there were more withdrawals and serious unwanted events with 300 mg. Without further research into the potential of optimal dosing, current evidence suggests bupropion might be second line for those failing with NRT or varenicline, although its cost-per-week benefit over these treatments is an advantage.

A potentially unique benefit for bupropion over NRT was our observation of a possible improvement in abstinence for those reporting a history of depression. This might be tested in future in a randomized controlled trial among those with a history of depression. Bupropion ameliorated the somewhat detrimental effect of depression observed with NRT, with a suggestion that when given bupropion those with depression, compared with those without, were more successful. Although this seems plausible, given bupropion's previous indication as an antidepressant, in three previous studies that compared bupropion with either placebo or low-dose bupropion no such additional benefit was observed among those with a history of depression 20–22. Taken together, these results would suggest that if bupropion is not additionally beneficial for those with a depression history, then NRT might at best be ineffectual, or even detrimental, for such smokers. Researchers with older data sets where depression history was recorded might investigate this. Perhaps surprisingly, our analysis of depression does not appear to have been undertaken in the three previous trials comparing bupropion with NRT 1–3. Given the high prevalence of depression among smokers seeking help to stop, our tentative finding merits further study 23.

### Clinical trial registration

International Standard Randomised Controlled Trial Number: ISRCTN91464711.

http://www.controlled-trials.com/ISRCTN91464711

### Declaration of interests

All authors have completed the Unified Competing Interest form at http://www.icmje.org/coi_disclosure.pdf (available on request from the corresponding author). R.W., P.H., H.M. and G.S. have acted as consultants to the manufacturers of nicotine replacement formulations, bupropion and varenicline, and have also given lectures sponsored by them. J.S. has acted as a consultant to Pfizer Ltd. J. Strang, Z.A., E.V., J.W., K.H., R.A. and C.O. report no financial relationships in connection with smoking.
